# Carbon Fiber-Reinforced Polyamide 6 Composites: Impact of Fiber Type and Concentration on the Mechanical Properties

**DOI:** 10.3390/ma18071413

**Published:** 2025-03-22

**Authors:** Weiping Dong, Zhaozhu Yu, Xingxiang Sun, Zhonglue Hu, Shiju E, Fangqiang Tong, Sisi Wang, Xiping Li

**Affiliations:** 1 Key Laboratory of Urban Rail Transit Intelligent Operation and Maintenance Technology & Equipment of Zhejiang Province, Zhejiang Normal University, Jinhua 321000, China; dwp@zjnu.cn (W.D.); yuzhaozhu@zjnu.edu.cn (Z.Y.); zhonglue.hu@zjnu.edu.cn (Z.H.); esx_2001@zjnu.cn (S.E.); 2 Zhejiang Baojing Carbon Materials Co., Ltd., Shaoxing 311800, China; sxx1465@vip.163.com (X.S.); tfqforever@163.com (F.T.); 3 Weida Cosmetics Packaging Industrial, Jinhua 321000, China

**Keywords:** polyamide 6, carbon fiber, mechanical properties, composite material

## Abstract

To investigate the influence of varying concentrations and types of carbon fiber (CF) on the mechanical properties of polyamide 6 (PA6) composites, this study explores the mechanical properties of PA6 composites with various CF types and quantities. The micro-morphology of the composites and the CF length distribution were characterized. The results indicate that the inclusion of carbon fibers significantly enhances the tensile, flexural, and notched impact strengths of PA6. Specifically, when about 30 wt% of CF T300 was added, the tensile and flexural strength of the composite reached a maximum of 166 MPa and 224 MPa, respectively, representing increases of 236.6% and 229.6%, respectively, compared to pure PA6. The maximum flexural modulus achieved 14.6 GPa, which was six times as large as that of pure PA6. Moreover, the CF length in the PA6 matrix follows a near-Gaussian distribution. A proper CF length and orientation, along with strong interfacial bonding between CF and the PA6 matrix, contribute to improved mechanical properties. The overall performance of T700-reinforced composites is better than that of T300-reinforced ones due to T700’s higher precursor strength and better fiber-length retention. This study provides guidance for fabricating high-performance PA6 composites.

## 1. Introduction

Carbon fiber (CF) has emerged as a revolutionary material in advanced manufacturing due to its exceptional combination of structural and functional properties. It demonstrates remarkable specific strength [1,2], with tensile strength reaching 3500–7000 MPa, while maintaining an ultra-low density of 1.5–2.0 g/cm^3^, approximately 60% lighter than aluminum alloys and 70% lighter than steel. This unique strength-to-weight ratio is complemented by outstanding wear resistance (coefficient of friction <0.2 under dry sliding conditions) and corrosion resistance that outperforms conventional metals in harsh environments, including saline and acidic media. Carbon fiber (CF)-reinforced polymer composite (CFRP) has numerous advantages, such as high strength, wear resistance, corrosion resistance, ease of processing, and thermal stability. As a result, it is extensively applied in the automotive, aerospace, medical devices, electronics, electrical, and mechanical sectors [3,4,5,6,7]. 

Polyamide 6 (PA6), as an essential polymer material, has a long-standing development history. Its performance and applications scope have been continually enhanced and refined with technological advancements. As the demand for improved performance grows, the demand for PA6 surges. CF outperforms other fibers in terms of specific strength and modulus, offering superior heat and corrosion resistance and friction resistance [8]. Thus, by combining carbon fiber with PA6 and harnessing their respective strengths for composite reinforcement, it is possible to create a new material that is strong, lightweight, and corrosion-resistant. Moreover, from the perspectives of environmental protection and sustainable development, research on carbon fiber-enhanced PA6 is of great significance. This material can replace traditional metal and alloy materials, reduce product weight, improve energy efficiency, and lower energy consumption in some fields. Its favorable recyclability and recourse utilization efficiency also contribute to the efficient use of resources and sustainable environmental development.

The interfacial bonding performance of carbon fiber and PA6 is crucial for the mechanical properties of composite components. Gon et al. [9] enhanced the interfacial bonding of PA6/CF composite by using a mixture of adhesive and reduced graphene oxide (RGO) coating, and the flexural strength and modulus of the modified composite increased by 73% and 84%, respectively. Li et al. [10] enhanced the interfacial adhesion between CF and PA6 matrix in PA6/CF composites by constructing a polydopamine/nano silica (PDA—SiO_2_) interface layer on the surface of carbon fibers. Compared with untreated PA6/CF, CF-PDA/PA6, and CF-SiO_2_/PA6 composites, the tensile strength of CF-PDA-SiO_2_/PA6 composites increased by 28.09%, 19.37%, and 26.22%, respectively. García Hernández et al. [11] reinforced PA6 composites with different contents of modified agave fibers (treated with 1% NaOH and ultrasonic waves) and characterized their structure, morphology, and mechanical properties. The interface interaction between agave fibers and PA6 functional groups led to an increase in Young’s modulus (18.8%), flexural strength (11.3%), and density (3.7%). Chenhui et al. [12] improved the interfacial adhesion between carbon fiber and matrix PA12 by CF oxidation and grafting PA6 onto the CF surface (noted as PA6-CF). The tensile strength of PA6-CF/PA12 composite increased from 53.9 MPa to 70.2 MPa, an increase of 30.8%. Zhu et al. [13] adjusted the interface properties by grafting graphene oxide (GO) onto the surface of CF with hyperbranched polyglycerol (HPG). After the introduction of GO at the interface, the shear strength of CF-reinforced PA6 and CF-reinforced epoxy composites increased by more than 44% and 30%, respectively. Sun et al. [14] generated a polydopamine (PDA) coating on CF by in situ synthesization of PDA coating on CF using ultrasound under anoxic conditions to modify the interface of the composite. The interlaminar shear properties of the PA6/CF composite modified by PDA increased by 21.07%. Fengnan et al. [15] studied the surface modification of regenerated carbon fiber (RCF) and its reinforcement effect on nylon 6 composite. Bisphenol A diglycidyl ether (DGEBA) was used as a coupling agent to modify the surface of RCF, which enhanced the interfacial adhesion between the fiber and nylon 6 matrix and improved the mechanical properties and thermal stability.

Furthermore, sizing agents exert a significant impact on the mechanical properties of PA6/CF composites. Zhao et al. [16] utilized an aqueous polyurethane sizing agent derived from natural biomass-based sodium alginate and investigated the effects of varying sizing agent concentrations on the PA6/CF composites, resulting in improved shear and flexural strengths. Gao et al. [17] developed a high solid-content hyperbranched waterborne polyurethane (HWPU) sizing agent and examined its influence on CF wettability and composite interfacial properties. Compared to the unmodified PA6/CF composites, the interlaminar shear and flexural strengths of the CF-1.5 wt%/PA6 composite increased by 50.3% and 27.7%, respectively, reaching 60.3 MPa and 508.4 MPa, respectively. Karsli et al. [18] investigated the influence of sizing agent types on the properties of CF-reinforced PA 6,6 composites. Both unsized and sized CF were utilized. Thermogravimetric analysis (TGA) revealed that polyurethane (PU) and PA sizing agents decomposed during processing. Mechanical and thermomechanical characterization demonstrated that the composites reinforced with sized CF exhibited higher tensile strength compared to those with unsized CF. Specifically, PA- and PU-sized CF composites achieved superior impact strength, storage modulus, and enhanced interfacial adhesion. Scanning electron microscopy (SEM) analysis further confirmed improved interfacial bonding between PU- or PA-sized CF and the PA 6,6 matrix. These results indicate that PA and PU are effective sizing agents for CF intended for use in PA 6,6-based composites.

Porosity and carbon fiber distribution, as key factors influencing the mechanical properties of composites, have attracted widespread attention. de Toro et al. [19] demonstrated that with the increment in CF content, the mechanical properties of the composite increased, although the porosity exhibited an upward trend. Usually, higher porosity may deteriorate the mechanical property. In their work, the impact of significantly increased CF content outweighed the slightly increased porosity. In terms of fiber length distribution, Karsli et al. [20] investigated the impact of fiber length and content on the mechanical, thermal, and morphological properties of CF-reinforced PA6 composites. The results indicate that as the CF content increases, the tensile strength, modulus, and hardness of the composites rise, while the fracture strain decreases. The melting enthalpy and relative crystallinity of the composites decrease with the increase in CF content. Conversely, the storage modulus and loss modulus of the composites increase with the increase in CF content. Cheng et al. [21] developed an overflow extrusion-annealing process for additive manufacturing, which significantly reduced the porosity of 3D-printed PA6-CF composites. Mechanical testing demonstrated that the rCF/PA6 composites fabricated using this method exhibited a tensile strength of 100.41 MPa (an 87.4% increase) and a flexural strength of 150.5 MPa (a 71% enhancement) compared to pure PA6.

However, there are limited studies on CF-reinforced PA6 composites of various CF types and quantities. To explore the impact of CF type and quantity on the mechanical properties of PA6 composites, a series of PA6 composites with various CF types and quantities were prepared in this work, and their mechanical properties were evaluated and analyzed. By comparing the effects of different CF types and quantities on the mechanical properties of composites, this study unveils the interfacial interaction mechanism between CF and PA6, thereby offering a theoretical grounding and practical guidance for the fabrication of high-performance CF-reinforced PA6 composites.

## 2. Materials and Methods

### 2.1. Materials

Polyamide 6 (PA6): YH, Hunan Yuehua Chemical Co., Ltd. (Hengyang, China). The density of pure PA6 is 1.13 g/cm^3^, and the melting temperature is 221.5 °C. T300 carbon fiber (CF): 12K unsized and sized; T700 carbon fiber: 12K unsized and sized (the grade of raw filament is different but the type of sizing agent is the same for T300 and T700 fibers, which is bisphenol A epoxy resin, and the content is about 1%), Zhejiang Baojing carbon materials Co., Ltd. (Shaoxing, China). The density of different CFs is characterized and shown in Table 1.

### 2.2. Preparation of PA6/CF Composites with Different Types and Contents of CF

**Figure 1:**
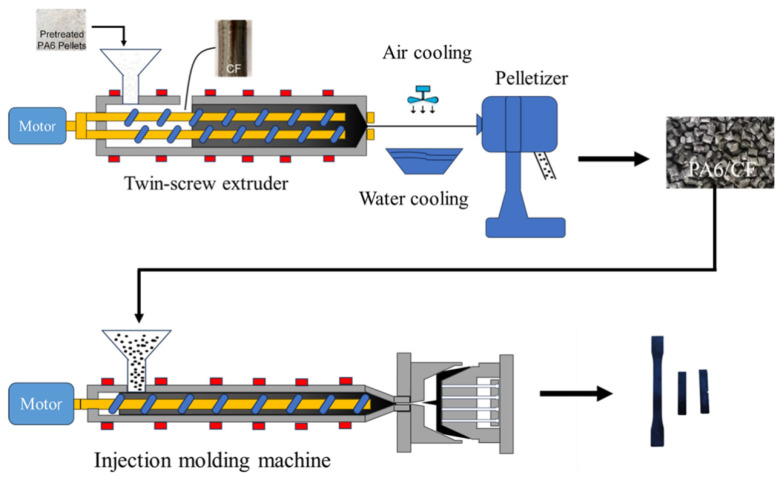
Schematic diagram of PA6/CF composite sample preparation.

Figure 1 illustrates the schematic diagram of the manufacturing process for PA6/CF composite particles. Pure PA6 particles and continuous CF were fed into the co-rotating parallel twin-screw extruder (model: SHJ-35A, produced by Nanjing Shengchi (Fuya) Rubber and Plastic Machinery Manufacturing Co., Ltd., Nanjing, China) and granulated. The temperature profile is 215, 220, 230, 240, 240, 240, 240, 245 °C; the screw speed of extruder is 300 rpm; and the feeding rate is 6 Hz (about 15 kg/h). Ten distinct PA6/CF composites were processed by introducing various types and numbers of continuous CF strands. The prepared composite particles were dried in an oven at 90 °C for more than 5 h before injection molding (Yizhimi plastic injection molding machine: FE120-430h, Guangdong Yizhimi Precision Machinery Co., Ltd., Foshan, China) at a temperature range of 235, 240, 240, 245, 250, 245, 240 °C to form standard specimen for tensile (ASTM D638-10, type I), flexural (ASTM D7264), and notched impact (ASTM D256) specimens. Because CF in the composite is incorporated in terms of strands, the actual CF content in the PA6/CF composite was measured (by thermogravimetric analyzer, TGA). The CF unsized (S0), one-strand carbon fiber sized (S1), two-strand carbon fiber sized (S2), three-strand carbon fiber sized (S3), and four-strand carbon fiber sized (S4) were added to PA6 and are named in Table 2.

### 2.3. Characterization

To investigate the influence of various CF types and concentrations on the mechanical properties of PA6/CF composites, 10 distinct tensile and flexural standard specimens were prepared and subjected to tensile testing at a rate of 25 mm/min (ASTM D638-10) using a universal testing machine (UTM4204, Shenzhen SUNS Technology Co., Ltd., Shenzhen, China), while flexural tests were conducted at a speed of 2 mm/min (ASTM D 7264). Impact standard samples underwent testing with a pendulum impact testing machine (PTM7151, Shenzhen SUNS Technology Co., Ltd.) under a pendulum energy of 5.5 J (ASTM D256). 

Scanning electron microscopy (SEM-30PLUS, Shanghai Oubotong Instrument Co., Ltd., Shanghai, China) was employed to scrutinize the notch impact fracture surface at an acceleration voltage of 20 kV. To enhance the quality of SEM observation, the fracture surface was sputter-coated with gold. 

To determine the actual CF content in the samples, 10 different granular specimens were heated from room temperature to 700 °C at a rate of 20 °C/min under nitrogen atmosphere using a thermogravimetric analyzer (TGA) (STA449F5, Netzsch, Saxony, Germany). To determine the CF length distribution in the samples, the thermogravimetric residue was put on a glass slide and examined using an inverted microscope (DMI3000 M, Leica, Wetzlar, Germany). Approximately 500 residual CFs were measured from the residues of the PA6/CF composites. 

Density measurements were conducted on the PA6/CF composite injection samples using an analytical balance (Sartorius Scientific Instruments Co., Ltd., Beijing, China).

Differential scanning calorimeter (Netzsch, Germany) test was performed to investigate the change in crystallinity with different CF types of fiber loading. A mass of about 10 mg was extracted from the injection sample and sealed in an aluminum crucible. The sample was heated from 30 °C to 260 °C and then cooled at a rate of 30 K/min under nitrogen atmosphere. The crystallinity ($X_{c}$) of samples was calculated according to the melting enthalpy observed in the first heating curve using the following formula:

(1) $$X_{c}=\frac{{∆H}_{m}}{{∆H}_{m}^{0}\times \omega }\times 100\%$$

where ${∆H}_{m}$ represents the melting enthalpy of the material, which is computed based on the melting peak value and is expressed in the unit of J/g; and ${∆H}_{m}^{0}$ denotes the melting enthalpy of the material at 100% crystallization with the unit of J/g. The theoretical melting enthalpy ${∆H}_{m}^{0}$ of PA6 is 230 J/g [22]; *ω* is mass percentage of PA6. The crystallization temperature observed in the cooling curve was recorded to compare the effect of CF type and content on the melt crystallization ability.

## 3. Results and Discussion

### 3.1. Mechanical Properties of PA6/CF Composites

**Figure 2:**
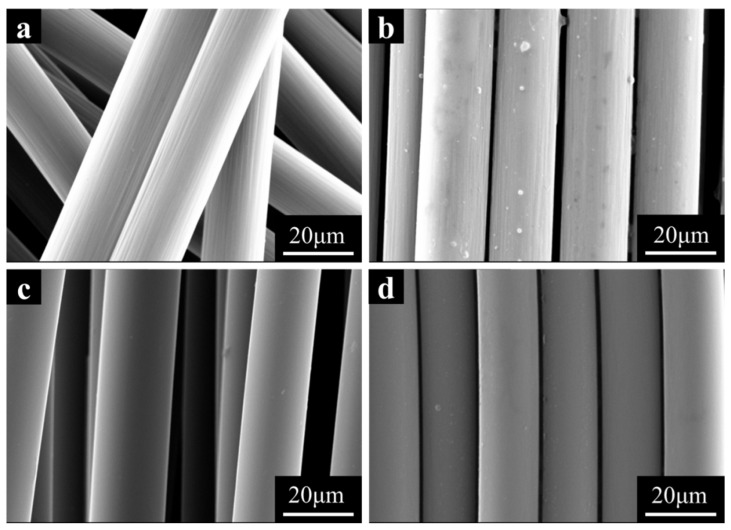
SEM of different CFs at 5000 times magnification. (**a**) T300 unsized fiber, (**b**) T300 sized fiber, (**c**) T700 unsized fiber, (**d**) T700 sized fiber.

In this study, pure PA6 was modified with T300 and T700 carbon fibers (unsized and sized). The surface of T300 and T700 carbon fibers have distinct characteristics, as depicted in Figure 2. This distinction is primarily attributed to the different spinning processes employed, with T300 manufactured via a wet spinning technique and T700 by utilizing a dry-jet wet spinning technique. The surface of T300 resembles bark and features channels of varying depths, whereas the surface of T700 carbon fiber is smoother. The sized CFs have a more orderly arrangement than the unsized fibers. Figure 2b shows the sized T300 fiber, which has a rougher surface compared to the unsized T300, which may be beneficial for the adhesion to the PA6 matrix and leads to better mechanical strength. While the surface roughness is similar for unsized and sized T700, the effect of the sizing agent on the mechanical performance will be discussed in the following section. T700 outperforms T300 in terms of tensile strength [23]. By measuring the actual CF content in PA6/CF composites with varying CF concentrations and evaluating their mechanical properties, we investigated the impact of different types and quantities of carbon fibers on the material’s mechanical properties.

**Figure 3:**
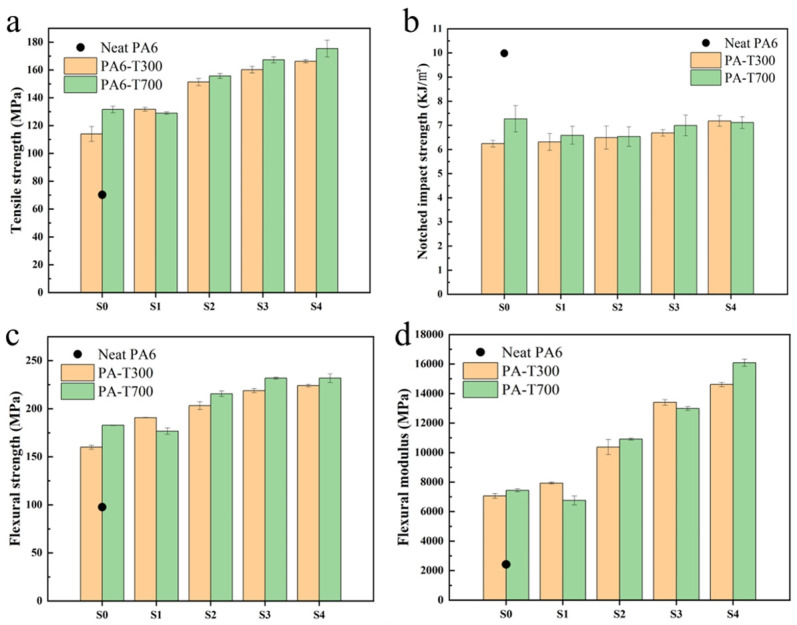
Mechanical properties of composites. (**a**) Tensile strength, (**b**) Notched impact strength, (**c**) Flexural strength, (**d**) Flexural modulus.

Based on the test results obtained from the universal testing machines and impact testing machines, the tensile strength, flexural strength, and notched impact strength of carbon fiber-reinforced PA6 composites with T300 and T700 series (S0 and S1, S2, S3, and S4) are presented in Figure 3.

Figure 3a shows that pure PA6 (the black point in Figure 3a) has the lowest tensile strength, approximately 70 MPa. All the composites with various CFs exhibit significantly higher tensile strength than pure PA6 (about 1.6–2.4 times higher). The tensile strength of PA6-T300-S0 is lower than that of PA6-T300-S1, which means that T300 CF unsized has a poorer reinforcement effect than T300 CF sized. With the increasing T300 CF, the tensile strength of the PA6 composite increases. Sample PA6-T300-S4 (when four strands of carbon fiber are added) has the highest tensile strength, reaching more than 160 MPa. 

The trend in the tensile strength of PA6 reinforced with different contents of T700 CF is similar to that of the composite reinforced with T300 CF, also showing an upward trend. The tensile strength of PA6-T700-S0 is slightly higher than that of PA6-T700-S1. As the CF content in T700 increases from S1 to S4, the tensile strength of the PA6/CF composites gradually increases. When the four strands of carbon fiber are added (denoted as S4), the tensile strength reaches the maximum, over 170 MPa. 

**Figure 4:**
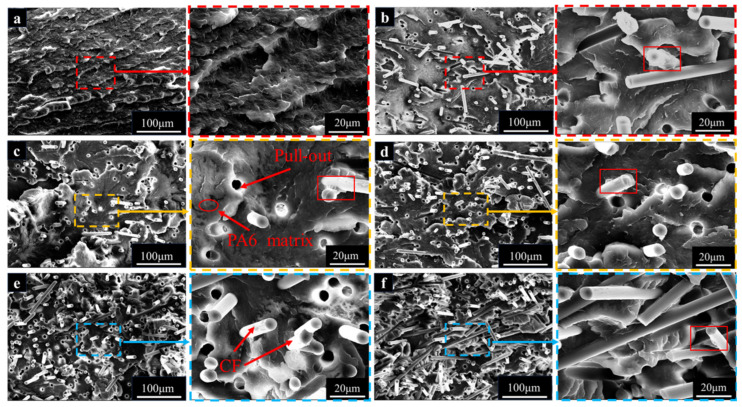
SEM image and enlarged view of impact sample cross-sections of PA6-T300 composites. (**a**) Pure PA6, (**b**) S0, (**c**) S1, (**d**) S2, (**e**) S3, (**f**) S4.

**Figure 5:**
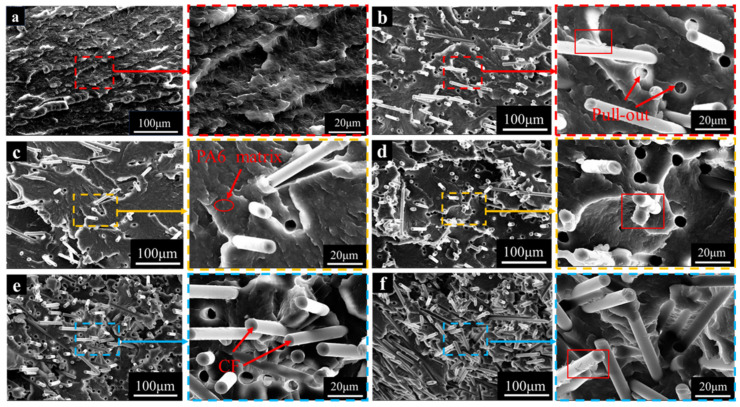
SEM image and enlarged view of impact sample cross-sections of PA6-T700 composites. (**a**) Pure PA6, (**b**) S0, (**c**) S1, (**d**) S2, (**e**) S3, (**f**) S4.

Generally, the tensile strength of PA6 reinforced with T700 CF is higher than that reinforced with T300 CF at varying CF contents. However, at a CF content of S1, the reinforcement effect of CF with T300 is superior to that of CF with T700. When referring to the micro-morphology of the composites (SEM images in Figure 4 and Figure 5), CF is integrated into the polymer and covered by the matrix, which facilitates load transfer to the CF and enhances the material’s tensile strength [24]. At a lower CF content, the primary fracture modes of the specimen are matrix fracture, along with CF debonding and pull-out. As the CF content increases, the tensile performance of the PA6/CF composites improves significantly. At a higher CF content, the fracture mode of the specimens mainly fractures through matrix fracture and CF fracture, resulting in relatively good tensile properties.

Figure 3b shows that the neat PA6 exhibits the highest notched impact strength, approximately 10.0 KJ/m^2^. When compared with pure PA6, the incorporation of CF reduces the notched impact strength of the composite. This reduction can be ascribed to stress concentration at the fiber ends, which initiates crack formation and subsequently causes the composite to fracture [25]. However, for PA6/CF composites with increasing CF T300 content, the notched impact strength increased gradually, reaching a maximum value of approximately 7.0 KJ/m^2^ at S4. By observing the SEM image of the fracture surface, it was found that the orientation of the detached carbon fibers forms a certain angle with respect to the impact cross-section, which can effectively inhibit crack propagation [24]. Moreover, the notched impact strength of PA6-T300-S0 is lower than that of PA6-T300-S1. 

The notched impact strength of PA6 reinforced with CFs of varying T700 content is similar to that of T300, also showing an upward trend. However, PA6-T700-S0 has the highest notched impact strength, reaching 7.3 KJ/m^2^. As the CF content in T700 increases from S1 to S4, the notched impact strength of PA6 gradually increases. The graph indicates that the overall impact strength enhancement of PA6 by T700 CF is slightly more significant than that of T300 CF.

Figure 3c clearly indicates that the flexural strength of pure PA6 without CF reinforcement is the lowest, merely about 100 MPa. In this figure, the flexural strength of PA6-T300-S0 is lower than that of PA6-T300-S1. For the T300 series, there is an upward trend in flexural strength with the increase in CF content, which indicates that the flexural strength of PA6 reinforced by T300 CF increases as the CF content rises [26]. Moreover, PA6/CF with S4 content has the highest flexural strength, reaching approximately 220 MPa. 

The overall trend of the PA6-T700 CF composites is similar to that of the T300 series, also presenting an upward trend. The flexural strength of PA6-T700-S0 is slightly higher than that of PA6-T700-S1. As the CF content in T700 increases from S1 to S4, the flexural strength of PA6 gradually rises, and the change in flexural strength of PA6 with a CF content between S3 and S4 tends to stabilize, reaching a maximum of about 230 MPa. Because CF has an extremely high tensile strength, it can absorb more flexural energy during mechanical deformation, thus contributing to an increase in the overall strength of the composite [27]. Generally, the flexural strength of PA6 reinforced with various contents of CF T700 outperforms that of T300.

Based on the analysis of Figure 3d, the flexural modulus of pure PA6 is the lowest, only about 2.4 GPa. The flexural modulus of PA6/CF reinforced with T300 CF increases as the CF content rises. The flexural modulus of PA6-T300-S0 is lower than that of PA6-T300-S1. As the CF content increases from S1 to S4, the flexural modulus of PA6 reinforced with T300 CF increases accordingly. When the CF content is at S4, the flexural modulus reaches the maximum, exceeding 14.0 GPa, which is nearly six times that of pure PA6. 

The overall trend of T700 in the figure is similar to that of T300, showing an increasing trend. The flexural modulus of PA6 gradually increases as the T700 CF content increases from S1 to S4. When the CF content is at S4, the flexural modulus reaches the maximum, approximately 16.4 GPa. Therefore, T700 CF has a better overall effect on the flexural modulus than T300 CF.

Overall, the incorporating of CFs (both T300 and T700) enhances the tensile and flexural strength of PA6. The overall performance of the T700 fiber-reinforced composites surpasses that of the PA6-T300 composites. This is attributed to the higher precursor strength of T700 and the better retention of longer fiber lengths within the matrix.

### 3.2. Microstructure of PA6/CF Composites

In the SEM image of the PA6-T300 sample group, the cross-section of PA-T300-S0 (Figure 4b) reveals that when pure PA6 is reinforced with unsized CF (S0), holes resulting from the pull-out of the CFs from the PA6 matrix can be observed, indicating the occurrence of CF debonding and pull-out. Comparing the PA6 matrices in Figure 4a,b, the pure PA6 matrix exhibits more folds, whereas the cross-section of the composite reinforced with S0 is smoother. When S0 is replaced with sized CFs (S1) as reinforcement, the amount of CF debonding and pull-out is similar to that in the case of S0 reinforcement. However, the folds in the PA6 matrix increase, which is consistent with the higher tensile strength of the PA6-T300 S1 sample compared to the S0 sample. 

In Figure 4c–f, with increasing CF content, more carbon fibers were observed. These fibers come into contact and interact with each other, leading to the clustering and interlacing of some fibers [28]. As the CF content rises, the circular gap left by CF pulling out increases, resulting in a rougher morphology of the impact section of the composite. The resin carried away by the pulled-out CFs is clearly visible within the red rectangular box, which indicates a favorable interface impregnation between the resin and the fibers [29]. It suggests that the degree to which the CFs are wrapped by the PA6 matrix has slightly improved, which corresponds to the enhancement of the mechanical properties of the composite with higher CF loading, as shown in Figure 3. Additionally, there is an increase in the wrinkling and deformation of the PA6 matrix, which implies that the CFs bear the primary load, thus improving the mechanical properties of the composites. 

When examining the cross-sections of the SEM images of the PA6-T700 composites (Figure 5), we observed a similar phenomenon to that of the PA6-T300 composites. As the T700 CF content increases, the tensile strength, notched impact strength, flexural strength, and flexural modulus of PA6/CF all increase. The trend in the mechanical properties in response to the change in CF content is similar to that of the T300 group. As the CF content rises, both the tensile and flexural strengths of T700-reinforced PA6 exceed those of T300-reinforced PA6, which is consistent with the fact that the T700 CF inherently has greater strength.

In the enlarged view of the PA6-T700 composite, when comparing the effect of the sizing agent, in Figure 5b,c, it is clear that the PA6 matrix encapsulates T700-S0 better than T700-S1. As a result, the mechanical properties of PA6-T700-S0 are superior to those of PA6-T700-S1. Similarly, the mechanical properties of PA6-T700-S0 outperform those of PA6-T300-S0. This is attributed to the fact that, under similar interfacial bonding conditions, the unsized T700 fiber has superior strength and modulus, which is in line with the mechanical property diagram (Figure 3). 

Upon examining Figure 5c–f, as the CF content rises, we observed that the circular pores left by the extraction of CF from the PA6 matrix also expanded. The extraction of CF brings along some resin, leading to an increase in the amount of CF with resin residue. This indicates that the resin achieves a good interfacial impregnation effect on the fibers. As a result, the degree to which the PA6 matrix encapsulates the CF is enhanced [30]. At a CF content of S4, CF achieves the most optimal interaction with the PA6 matrix. 

With an identical screw configuration and processing technique, the surface of T300 CF (Figure 2b) is rougher than that of T700 CF (Figure 2d). When subjected to external forces, the adhesion between T300 CF and the PA6 matrix outperforms the adhesion between T700 CF and PA6, leading to improved comprehensive mechanical properties of PA-T300 S1 compared to PA-T700 S1. However, when comparing the cross-sectional profiles of samples from the PA6-T300 and PA6-T700 groups (Figure 4 and Figure 5), the cross-section of the PA6-T700 group samples is smoother. This is attributed to the flaking of the matrix surface, primarily caused by long carbon fibers. 

**Figure 6:**
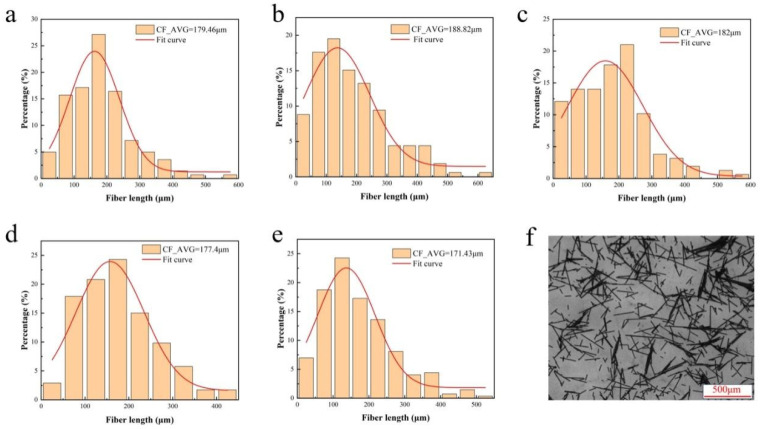
PA6-T300-CF length distribution. (**a**) PA6-T300-S0, (**b**) PA6-T300-S1, (**c**) PA6-T300-S2, (**d**) PA6-T300-S3, (**e**) PA6-T300-S4, (**f**) Optical microscope image of CFs in the residual PA6-T300-S2 sample by thermogravimetry.

**Figure 7:**
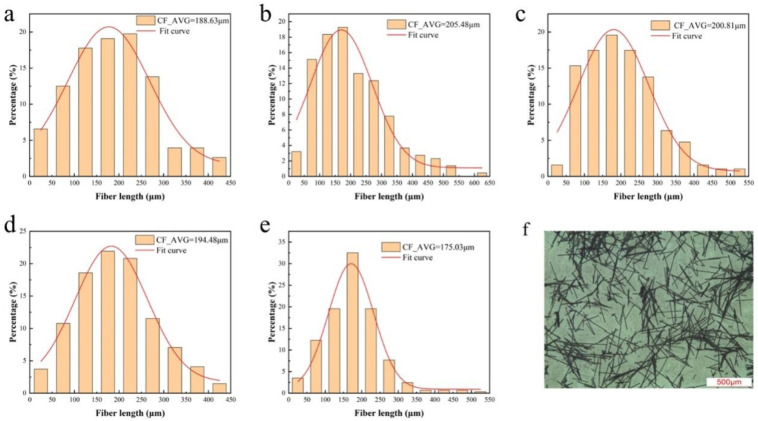
PA6-T700-CF length distribution. (**a**) PA6-T700-S0, (**b**) PA6-T700-S1, (**c**) PA6-T700-S2, (**d**) PA6-T700-S3, (**e**) PA6-T700-S4, (**f**) Optical microscope image of CFs in the residual PA6-T700-S2 sample by thermogravimetry.

**Figure 8:**
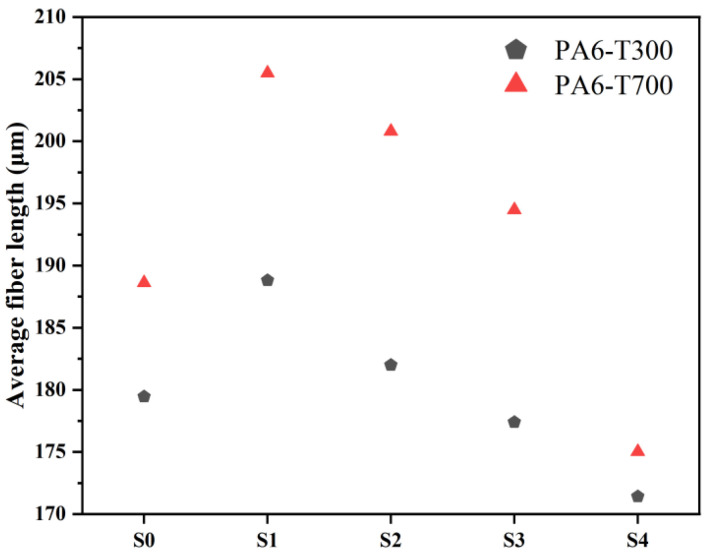
Average length of CFs in each composite material.

To evaluate the length distribution of CFs in PA6/CF composites, the residues of PA6/CF composites after complete thermogravimetric burning were observed using an inverted microscope. The length distribution of CFs in each composite material is shown in Figure 6 and Figure 7. The average length of the CFs in each composite is summarized in Figure 8.

Figure 6 shows that sized CF (S1) has a longer residual fiber length compared to the unsized one (S0). As the CF content increases, the average length of the CFs in PA6/CF T300 decreases. At 10 wt% CF content (S0 and S1), the increase in the average CF length indicates that long fibers contribute to enhancing the mechanical properties of the composite [31]. Subsequently, the decrease in average length can be attributed to the excessive CF content during melt blending and extrusion, leading to greater interactions among the fibers, thereby increasing the likelihood of fiber fracture [20]. As a result, the average length is reduced. However, the enhancement of the mechanical properties due to the high CF content outweighs the effect of the shorter fiber length. 

Similarly, Figure 7 shows that the sized CF (S1) has a longer length in PA6/CF than the unsized one (S0). With increasing T700 CF content, the average CF length decreases. Notably, as depicted in Figure 8, the average length of the fibers in PA6 reinforced with T700 is slightly longer than that in PA6 reinforced with T300. This is because T300 fibers have a rougher surface than T700 fibers, which causes them to experience greater shear and forces during melt blending. Consequently, they have a shorter average length. The longer length of the carbon fibers can be attributed to the higher mechanical properties of PA6-T700 composites compared to PA6-T300 (Figure 3). 

Overall, the distribution of CF lengths in the composites follows a classic normal distribution. The majority of the fibers (>60%) have lengths between 100 μm and 300 μm. Moreover, we observe ultra-long fibers (>500 μm), with the longest exceeding 500 μm. Preserving such long fibers is beneficial for achieving high mechanical strength [32].

In general, with increasing CF loading, SEM images show denser fibers, more fiber pull-out, and rougher fracture surface, indicating improved mechanical properties. The CF type with higher inherent modulus (T700) resulted in higher reserved length and higher mechanical properties. Interestingly, although the CF length reduced with higher CF loading, CF content plays a more important role in mechanical performance compared to CF length. 

### 3.3. Thermogravimetric Analysis of PA6/CF Composites

**Figure 9:**
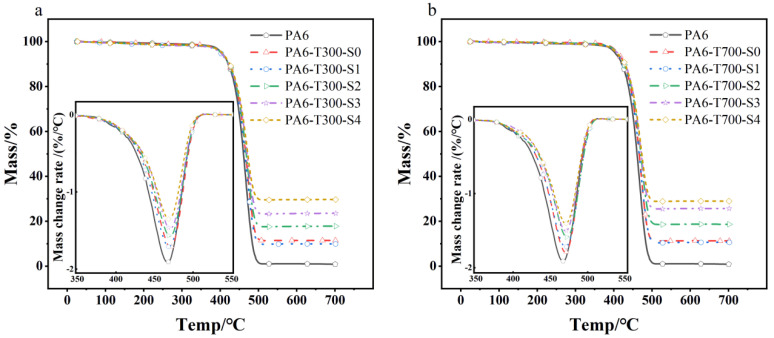
TG and inserted DTG curves of different content and types of CF-reinforced PA6 composites. (**a**) PA6-T300 composites, (**b**) PA6-T700 composites.

Thermal stability is a crucial characteristic of polymer composites. Figure 9 shows the TG and DTG curves for various CF contents and type PA6/CF composites. The data in Figure 9a indicate that as the CF content of different types increases, there is no significant change in the decomposition temperature of the composites. Table 3 summarizes the findings, revealing that the initial decomposition temperature of the composites is approximately 403 °C, with the maximum decomposition temperature around 470 °C. While pure PA6 decomposes completely, leaving behind minimal residues (particularly, with the lowest mass fraction of approximately 0.88%), the PA6 composites with S4 have the highest mass fraction of complete decomposition residues, approximately 28%. This trend continues to rise as the CF content increases.

Based on the data presented in Figure 9 and Table 3, it is evident that at the point of reaching the maximum thermal decomposition rate, pure PA6 exhibits the lowest temperature of maximum weight loss, at approximately 465 °C. By contrast, the maximum weight loss rate temperatures for PA6/CF composites with various grades of CF are all around 470 °C. This suggests that CF reinforcement of PA6 can marginally raise the composite’s maximum weight loss rate temperature, but the CF content has a minimal influence on this temperature. 

As expected, the density of the PA6/CF composites increases as the CF content rises. Notably, the pure PA6 sample has the lowest density, at 1.13 g/cm^3^. With the highest CF content (S4), the density of the composites reaches a maximum of 1.26 g/cm^3^. There is a minimal difference in density between the T300 CF- and T700 CF-reinforced PA6 composites. The peak value on the DTG curve rises slightly with increasing CF content, indicating a somewhat enhanced thermal stability of the composites [24].

### 3.4. DSC Analysis

**Figure 10:**
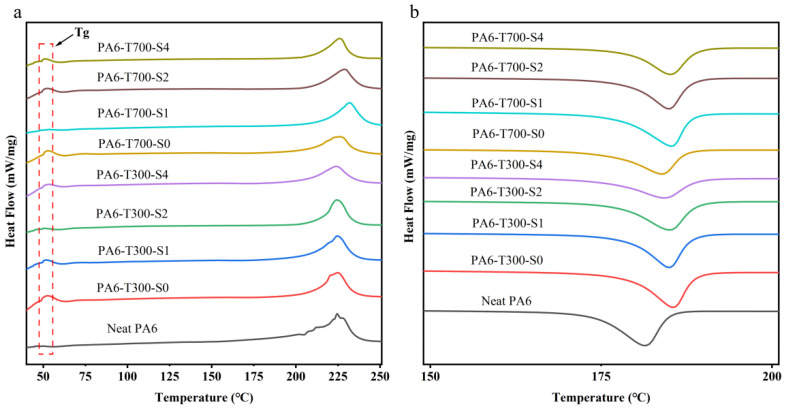
The (**a**) first heating and (**b**) cooling DSC curves of PA6 composite materials.

Figure 10 shows the DSC melting curves of PA6/CF composites with various CF contents. Apparently, in Figure 10a, the melting temperature of pure PA6 is approximately 224 °C, and the addition of CF has a minimal impact on that of the PA6/CF composites. The glass transition temperature (Tg) of the composites is around 50 °C and remains relatively stable with the increasing CF content. The melting enthalpy of pure PA6 is 103 J/g, while that of the CF-filled PA6 composite stays relatively constant at around 50 J/g (as depicted in Table 4). 

By analyzing the data in Table 4, it is evident that the crystallinity of pure PA6 is 44.83%, and the addition of CF decreases the crystallinity of the PA6/CF composite. The carbon fiber loading has a negligible effect on the crystallinity of the composite; the crystallinity of all the PA6-CF composites is about 30%. Acting as a heterogeneous nucleating agent, CF augments the number of nucleation sites and accelerates the nucleation rate. Conversely, CF impedes the crystal growth process, and the growth rate fails to match the nucleation rate, leading to the formation of smaller and imperfect crystals and, ultimately, a reduction in crystallinity [10].

In Figure 10b, notably, the crystallization temperature of the CF/PA6 composite is slightly higher (around 185 °C) than that of PA6 (181.4 °C), signifying that CF accelerates the crystallization ability of PA6, lowers the energy required for crystallization, and elevates the crystallization temperature. 

To sum up, the addition of CF reduces the crystallinity of PA6, although it enhances the crystallization rate. The loading has a rare effect on the crystallization behavior of PA6.

## 4. Conclusions

This study investigates the influence of different CF types and concentrations on the mechanical properties of PA6/CF composites. Ten groups of composites were prepared with varying CF types and concentrations, and their mechanical properties were evaluated. 

The results demonstrate that the incorporating of carbon fiber (both T300 and T700) enhances the tensile and flexural strength of PA6. As the CF content increases, both tensile and flexural properties gradually improve. And, the addition of four strands of CF yields the highest performance. Specifically, the tensile and flexural strengths of the PA6/T300-S4 composite are 166 MPa and 224 MPa, respectively, representing increases of 236.6% and 229.6%, respectively, compared to pure PA6. The maximum flexural modulus reaches 14.6 GPa, indicating a 602.9% increase over that of pure PA6. The notched impact strength of the composite is lower than that of pure PA6, but it shows an overall upward trend as the CF content increases. The maximum notched impact strength is 7.18 kJ/m^2^, which is 28.1% lower than that of pure PA6.

The overall performance of the T700 fiber-reinforced composite produced via the dry process exceeds that of the PA6-T300 composite. This superior performance is mainly attributed to the higher precursor strength and the retention of longer fiber lengths of T700 in the matrix. The sizing agent has different influences on T300 and T700 fibers. For T300 CF, fibers coated with a sizing agent help protect the fibers and enhance their mechanical properties. The T300-S1 sample is less affected by screw shear, which results in a longer fiber length and superior mechanical properties compared to the T300-S0 sample without sizing agent modification. However, the sizing agent has a minimal impact on PA6-T700. Due to the high precursor modulus of T700 and its smooth fiber surface both before and after sizing agent modification, the mechanical properties of the T700-S1 and T700-S0 samples are comparable, showing no significant difference.

The type and content of CF are key factors affecting the mechanical properties of PA6 composites. In practical applications, the appropriate type and content of CF should be selected according to specific needs, and the mechanical properties of composites should be improved through appropriate treatment methods. This study provides a useful reference and guidance for the performance optimization and application expansion of PA6 composites.

## Figures and Tables

**Table 1:** Density of different CFs.

Model	Density (g/cm^3^)
T300-CF unsized	1.57
T300-CF sized	1.56
T700-CF unsized	1.77
T700-CF sized	1.62

**Table 2:** Names of 10 kinds of PA6/CF composites and their actual content of CF obtained by thermogravimetric analysis.

Model	Name	CF Actual Content
PA6-T300-CF unsized	PA6-T300-S0	11.46 wt.%
PA6-T300-1 strand CF sized	PA6-T300-S1	9.94 wt.%
PA6-T300-2 strand CF sized	PA6-T300-S2	17.78 wt.%
PA6-T300-3 strand CF sized	PA6-T300-S3	23.54 wt.%
PA6-T300-4 strand CF sized	PA6-T300-S4	29.66 wt.%
PA6-T700-CF unsized	PA6-T700-S0	11.26 wt.%
PA6-T700-1 strand CF sized	PA6-T700-S1	10.62 wt.%
PA6-T700-2 strand CF sized	PA6-T700-S2	18.65 wt.%
PA6-T700-3 strand CF sized	PA6-T700-S3	25.64 wt.%
PA6-T700-4 strand CF sized	PA6-T700-S4	28.98 wt.%

**Table 3:** Pyrolysis process of PA6/CF composites with different CF contents at the same heating rate.

Material Name	Temperature at 5% Decomposition (°C)	TG Residue (wt.%)	Maximum Weight Loss Rate Temperature (°C)	Density (g/cm^3^)
Neat PA6	403.11	0.88	465.04	1.13
PA6-T300-S0	402.23	11.46	471.09	1.15
PA6-T300-S1	400.34	9.94	468.32	1.17
PA6-T300-S2	402.25	17.78	470.19	1.18
PA6-T300-S3	400.17	23.54	470.22	1.23
PA6-T300-S4	404.53	29.66	470.44	1.26
PA6-T700-S0	410.37	11.26	470.35	1.16
PA6-T700-S1	406.32	10.62	468.32	1.16
PA6-T700-S2	408.57	18.65	472.45	1.19
PA6-T700-S3	409.70	25.64	470.58	1.22
PA6-T700-S4	407.14	28.98	473.14	1.26

**Table 4:** The data for the first heating curve of DSC.

Sample Code	T_m_ (°C)	T_c_ (°C)	∆H_m_ (J/g)	X_c_ (%)
Neat PA6	224.60	181.40	103.10	44.83
PA6-T300-S0	224.90	185.60	61.85	30.37
PA6-T300-S1	224.70	185.00	66.21	31.96
PA6-T300-S2	224.20	185.00	55.78	29.50
PA6-T300-S4	224.00	184.20	49.97	30.89
PA6-T700-S0	226.30	183.90	53.89	26.40
PA6-T700-S1	229.40	184.30	62.12	30.22
PA6-T700-S2	229.00	184.90	53.71	28.71
PA6-T700-S4	226.00	185.20	45.54	27.88

## Data Availability

The original contributions presented in this study are included in the article. Further inquiries can be directed to the corresponding authors.
